# SLA-MLP: Enhancing Sleep Stage Analysis from EEG Signals Using Multilayer Perceptron Networks

**DOI:** 10.3390/diagnostics14232657

**Published:** 2024-11-25

**Authors:** Farah Mohammad, Khulood Mohammed Al Mansoor

**Affiliations:** 1 Department of Computer Science and Technology, Arab East Colleges, Riyadh 11583, Saudi Arabia; 2 Self-Development Skills Department, King Saud University, Riyadh 11543, Saudi Arabia; kalmansoor@ksu.edu.sa

**Keywords:** sleep disorder, data balancing, classification, EEG Signals, TCN, MLP

## Abstract

**Background/Objectives**: Sleep stage analysis is considered to be the key factor for understanding and diagnosing various sleep disorders, as it provides insights into sleep quality and overall health. **Methods**: Traditional methods of sleep stage classification, such as manual scoring and basic machine learning approaches, often suffer from limitations including subjective biases, limited scalability, and inadequate accuracy. Existing deep learning models have improved the accuracy of sleep stage classification but still face challenges such as overfitting, computational inefficiencies, and difficulties in handling imbalanced datasets. To address these challenges, we propose the Sleep Stage Analysis with Multilayer Perceptron (SLA-MLP) model. **Results**: SLA-MLP leverages advanced deep learning techniques to enhance the classification of sleep stages from EEG signals. The key steps of this approach include data collection, where diverse and high-quality EEG data are gathered; preprocessing, which involves signal cropping, spectrogram conversion, and normalization to prepare the data for analysis; data balancing, where class weights are adjusted to address any imbalances in the dataset; feature extraction, utilizing Temporal Convolutional Networks (TCNs) to extract meaningful features from the EEG signals; and final classification, applying a Multilayer Perceptron (MLP) to accurately predict sleep stages. **Conclusions:** SLA-MLP demonstrates superior performance compared to traditional methods by effectively addressing the limitations of existing models. Its robust preprocessing techniques, advanced feature extraction, and adaptive data balancing strategies collectively contribute to obtaining more accurate results, having an accuracy of 97.23% for the S-DSI, 96.23 for the S-DSII and 97.23% for the S-DSIII dataset. This model offers a significant advancement in the field, providing a more precise tool for sleep research and clinical applications.

## 1. Introduction

Sleep disorders are conditions that disrupt normal sleep patterns, affecting the quality, duration, or timing of sleep [1]. These disorders include a range of issues such as insomnia, which involves difficulty falling or staying asleep; sleep apnea, characterized by interruptions in breathing during sleep; restless legs syndrome, which causes uncomfortable sensations in the legs and an urge to move them; and narcolepsy, marked by sudden and uncontrollable sleep attacks. The effects of sleep disorders are profound and multifaceted, impacting both physical and mental health. Individuals with sleep disorders often experience chronic fatigue, reduced cognitive function, and mood disturbances, including increased irritability and anxiety [2]. Long-term sleep disturbances can also lead to serious health issues such as cardiovascular diseases, obesity, diabetes, and weakened immune function. The pervasive nature of sleep disorders can significantly impair daily functioning and overall quality of life, underscoring the importance of effective diagnosis and treatment [3].

Sleep stage analysis is vital for diagnosing and managing sleep disorders by providing detailed insights into the various phases of sleep and their quality [4]. By examining different sleep stages—such as wakefulness, light sleep, deep sleep, and REM sleep—healthcare professionals can pinpoint disruptions and abnormalities, such as frequent awakenings or insufficient deep sleep, which are indicative of conditions like sleep apnea or insomnia. This analysis enables accurate diagnosis and allows for the development of personalized treatment plans tailored to specific sleep issues. Additionally, it helps in monitoring the effectiveness of treatments by offering objective data on improvements or ongoing challenges [5]. Furthermore, sleep stage analysis supports research and development by contributing to the understanding of sleep disorders and the creation of new diagnostic tools and therapies. In essence, it enhances the ability to diagnose, treat, and manage sleep disorders effectively, thereby improving overall sleep health and quality of life.

Traditional methods of Sleep Stage Analysis (SSA) primarily include manual scoring and basic automated systems [6]. Manual scoring, often performed through polysomnography (PSG), involves trained technicians visually inspecting sleep recordings and assigning sleep stages based on established criteria. While this method provides detailed information, it is labor-intensive, time-consuming, and prone to human error and subjectivity [7]. Automated systems, which use simple algorithms to classify sleep stages based on features extracted from EEG signals, offer a more scalable approach but often face limitations such as insufficient accuracy, inability to handle noisy or complex data, and difficulties in adapting to individual variations in sleep patterns. Both methods are challenged by issues like the inability to capture subtle sleep stage transitions and the inefficiency of processing large volumes of data. Consequently, these traditional methods may lead to inaccurate diagnoses and less effective management of sleep disorders, highlighting the need for more advanced, reliable techniques in sleep stage analysis.

Computer-based solutions such as machine learning and deep learning-based solutions have significantly advanced the field of sleep stage analysis by automating and enhancing the accuracy of sleep stage classification [8]. Machine learning approaches, such as support vector machines (SVMs) and random forests, utilize features extracted from EEG signals to classify sleep stages based on pre-defined patterns [9]. These methods can improve accuracy and scalability compared to traditional techniques but often struggle with limitations such as overfitting, requiring extensive feature engineering, and difficulties in generalizing to diverse datasets.

Deep learning approaches, including convolutional neural networks (CNNs) and recurrent neural networks (RNNs), have further enhanced sleep stage classification by learning hierarchical features directly from raw EEG data [10]. These models can capture complex patterns and improve accuracy by automatically identifying relevant features without extensive manual intervention. Despite their advantages, deep learning methods face challenges such as high computational costs, the need for large amounts of labeled data for training, and potential difficulties in interpreting model decisions. Additionally, deep learning models can be prone to overfitting if not properly regularized and may struggle with class imbalance or noisy data. Overall, while machine learning and deep learning solutions offer significant improvements, they are not without their limitations, and ongoing research is needed to address these challenges and further advance the field.

The Sleep Stage Analysis based on SLA-MLP represents a significant advancement over existing sleep stage classification methods by addressing key limitations of traditional and current deep learning approaches. Unlike manual scoring and basic automated systems, SLA-MLP integrates advanced preprocessing techniques, robust data balancing strategies, and sophisticated feature extraction to provide a more accurate and reliable classification of sleep stages. By leveraging Temporal Convolutional Networks (TCNs) for feature extraction and employing a Multilayer Perceptron (MLP) for final classification, SLA-MLP effectively captures complex patterns in EEG data while mitigating issues such as overfitting and class imbalance. Its ability to handle diverse datasets with greater precision and efficiency highlights its superiority over traditional machine learning methods and other deep learning models. Consequently, SLA-MLP offers a more comprehensive and scalable solution for sleep stage analysis, promising improved diagnostic accuracy and better management of sleep disorders.

### Research Contribution

The proposed SLA-MLP model contributes significantly to sleep stage classification through the following:

- Utilizing Temporal Convolutional Networks (TCNs) for feature extraction, SLA-MLP effectively captures complex temporal patterns in EEG signals, enhancing feature quality and classification accuracy;
- The Multilayer Perceptron (MLP) used for classification offers advanced non-linear capabilities, improving the differentiation between sleep stages and enhancing mental health;
- SLA-MLP achieves impressive accuracy rates of 97.23% for S-DSI, 96.23% for S-DSII, and 97.23% for S-DSIII, demonstrating its robustness and effectiveness in sleep stage analysis

The remainder of this paper is organized as follows: Section 2 reviews current techniques for sleep stage classification, Section 3 describes the methodology of the proposed approach, Section 4 presents the experimental results and evaluations, and Section 5 concludes with a discussion and suggestions for future research.

## 2. Literature Review

Recent advancements in sleep stage classification have demonstrated significant progress through the application of machine learning and deep learning techniques. Traditional methods often struggle with limitations such as restricted generalizability, high computational demands, and black-box behavior that impedes clinical use. Pradeepkumar et al. [11], present a novel approach to sleep stage classification by introducing a cross-modal transformer. They address a critical limitation of current deep-learning models—their black-box behavior—which restricts their use in clinical settings. The proposed model incorporates a transformer-based architecture combined with a multi-scale one-dimensional convolutional neural network for automatic feature representation. This approach not only achieves state-of-the-art performance but also enhances interpretability through attention modules, providing significant reductions in parameters and training time. The study’s findings underscore the potential of combining advanced models to improve both accuracy and clinical applicability.

Zao et al. [12] focus on leveraging temporal context to enhance sleep stage classification. They identify a gap in existing multi-task learning methods, which often ignore the importance of sequential temporal context for capturing long-term dependencies. To address this, they propose a multi-task deep learning model that simultaneously reconstructs sequence signals and segments time series. The model employs a one-dimensional channel attention module to refine feature representation, leading to improved performance in single-channel EEG analysis. Their experimental results across four datasets reveal competitive accuracy, highlighting the effectiveness of their approach in capturing the sequential nature of sleep stages.

Guo et al. [13] presented a deep learning model based on the residual network (ResNet) architecture, designed for classifying sleep states using both EEG and EMG signals. By leveraging residual connections, the model effectively handles the complexities of sleep data and achieves high accuracy in sleep stage classification. The research demonstrates the model’s robustness and efficiency, with results showing notable performance improvements over traditional methods. The approach highlights the benefits of using advanced neural network architectures to improve classification accuracy and operational efficiency.

Vaquerizo-Villar et al. [14] proposed a convolutional neural network (CNN) methodology for classifying sleep stages from EEG signals. Their approach involves segmenting EEG signals into 30 s epochs, converting these into 2D time–frequency representations, and applying a CNN for classification. The model achieves high accuracy, with exceptional performance reported for various channels. This method underscores the potential of CNNs to improve sleep stage classification by leveraging detailed time–frequency information from EEG signals, though it also points to the need for efficient computational strategies.

Li et al. [15] explore an autoencoder-based unsupervised learning approach for sleep stage classification. This methodology focuses on feature extraction and dimensionality reduction without requiring labeled data. The autoencoder model demonstrates effectiveness in learning useful representations from sleep data, providing insights into sleep stages with reduced reliance on manual annotations. Despite its advantages, the approach faces challenges related to noise and the interpretability of learned features, which can impact its practical application.

Toma et al. [16] designed a model combining Temporal Convolutional Networks (TCNs) and Multilayer Perceptrons (MLPs) for sleep stage classification. This hybrid approach leverages the temporal feature extraction capabilities of TCNs and the classification strengths of MLPs. The model achieves high accuracy across diverse datasets, demonstrating its effectiveness in handling complex sleep data. The integration of TCNs with MLPs offers a robust solution for improving classification performance while addressing some limitations of previous models, such as computational efficiency and accuracy. The some of existing approaches are shown in Table 1. 

From the above discussion it has been concluded that there are several limitations in existing sleep stage classification methodologies, including restricted generalizability due to dataset limitations, high computational costs, and the need for extensive tuning. Traditional models often rely on specific datasets or channels, which can limit their applicability across different contexts and require significant computational resources. Additionally, some approaches suffer from reduced accuracy when dealing with noisy or incomplete data, and their complex architectures can hinder practical implementation. In contrast, the SLA-MLP model addresses these challenges effectively by leveraging a sophisticated combination of Temporal Convolutional Networks (TCNs) and a Multilayer Perceptron (MLP) for feature extraction and classification. SLA-MLP demonstrates superior performance with high accuracy across diverse datasets, providing a more scalable and efficient solution for sleep stage analysis. This model not only enhances classification accuracy but also reduces the computational burden compared to traditional methods, making it a valuable advancement in the field of sleep research.

## 3. Proposed Methodology

**Figure 1:**
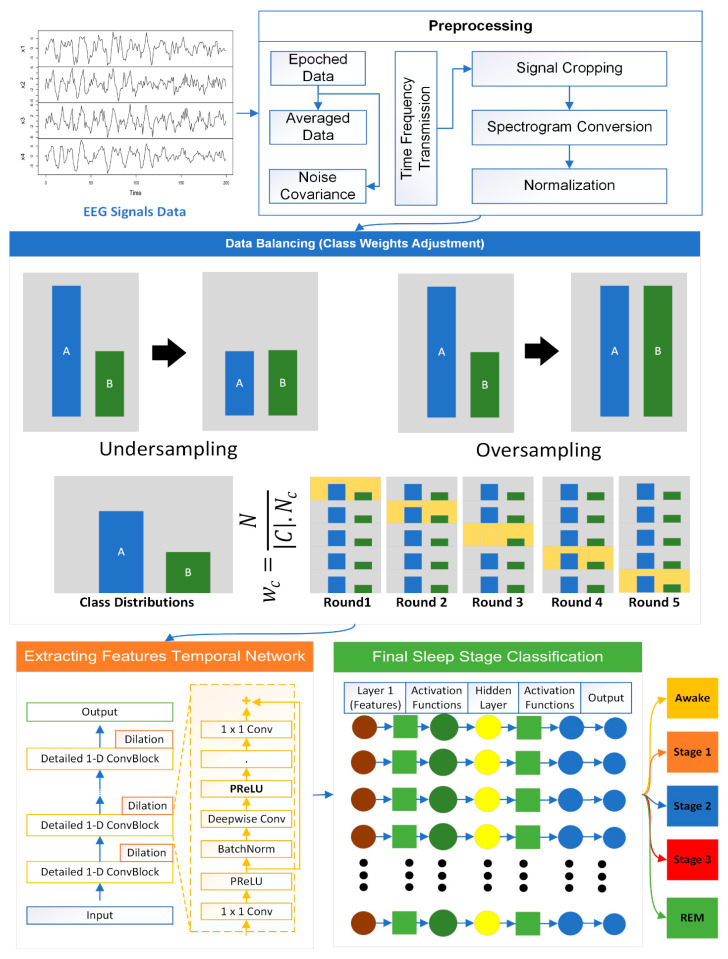
Proposed SLA-MLP model.

This section discusses the proposed architecture mentioning the workflow of all steps as shown in Figure 1. The proposed methodology consists of data collection, followed by data preprocessing and feature extraction. Once features are extracted, the data augmentation is applied. The deep learning model is then trained on the training data. The proposed model is then evaluated on performance matrices, and lastly, post processing is applied.

### 3.1. Data Collection

The first dataset abbreviated as S-DSI is obtained from the Sleep-EDF Database, a cornerstone database in sleep research, providing polysomnographic recordings from 20 healthy Caucasian subjects aged 25–34. The dataset includes 153 whole-night recordings, each typically around 8 h long, with EEG sampled at 100 Hz. It contains annotations for sleep stages (W, N1, N2, N3, and REM) based on the Rechtschaffen and Kales (R&K) standard. The dataset offers a rich source of data for sleep stage classification, with a total of approximately 1.4 million 30 s epochs labeled across the recordings. The data include EEG from two channels, Fpz-Cz and Pz-Oz, and are available on PhysioNet. The next dataset is S-DSII which is obtained from the MASS Dataset, which is an extensive collection of polysomnographic recordings from 200 subjects, providing a robust resource for sleep research. This dataset features multiple sleep sessions per subject, with a total of 579 recordings. EEG data are recorded at 256 Hz, and the dataset includes detailed sleep stage annotations following both the R&K and AASM (American Academy of Sleep Medicine) standards. The MASS dataset covers five EEG channels, including C4-A1 and F4-C4, with each recording averaging 7–8 h. It encompasses over 2 million 30 s epochs, offering a diverse range of sleep patterns for in-depth analysis and model training.

The final dataset is S-DSIII which is gathered from the SHHS Dataset, which is one of the largest sleep datasets available, encompassing polysomnographic recordings from 6441 subjects aged 40 and older. The data include full-night recordings with EEG sampled at 125 Hz and annotations for sleep stages (W, N1, N2, N3, REM) based on the AASM standard. The dataset provides an invaluable resource for studying sleep-disordered breathing, with over 51,000 h of sleep data and approximately 6.1 million 30 s epochs labeled. It includes EEG data from the C3-A2 channel and offers a broad demographic representation, making it a critical dataset for exploring sleep patterns across different populations.

**Figure 2:**
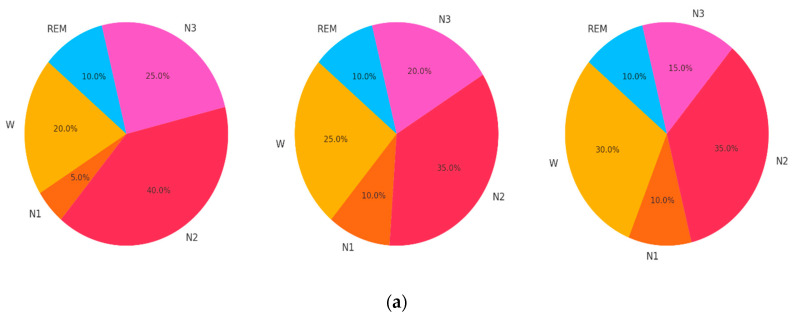

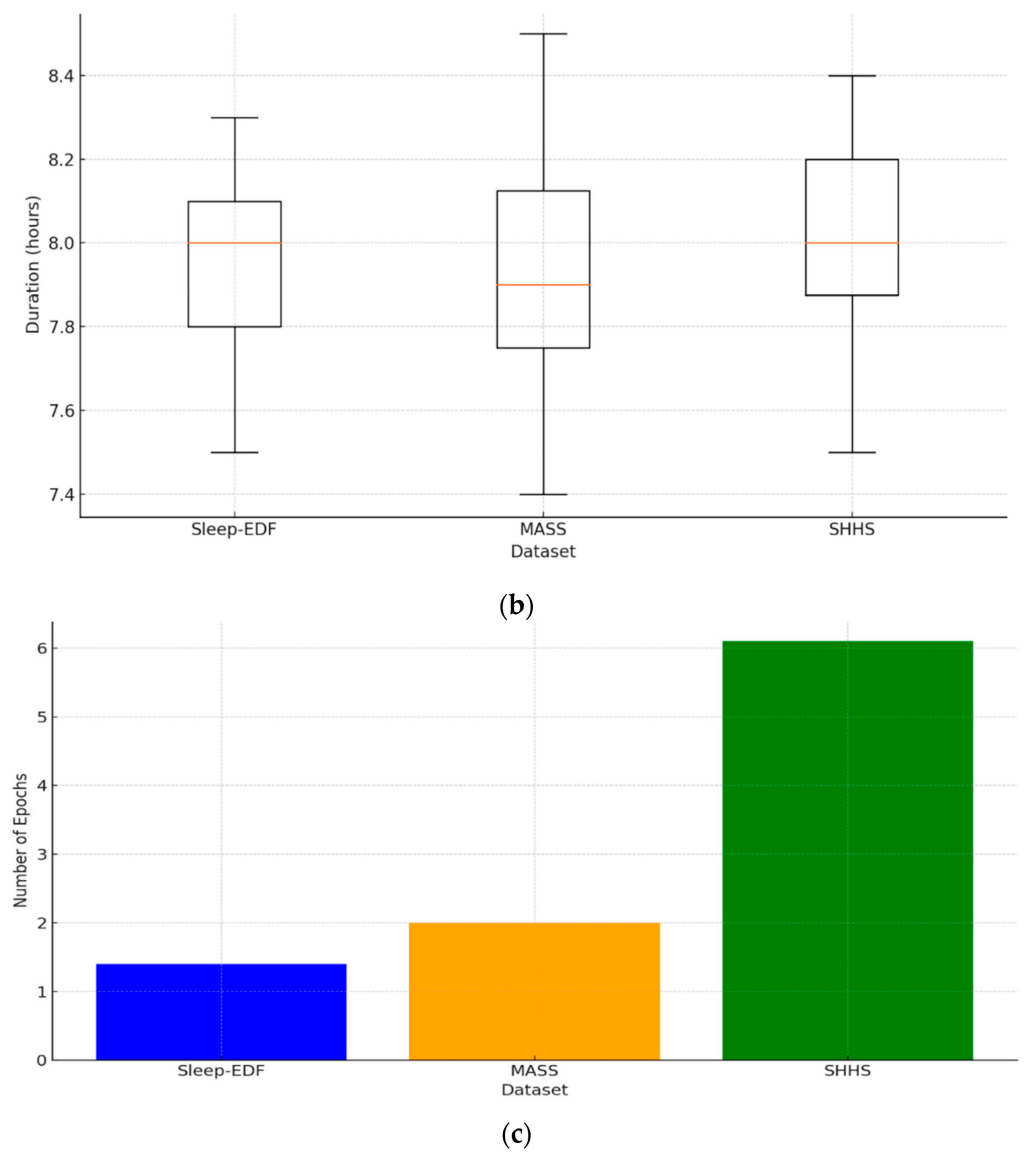
Dataset description. (**a**) Sleep stage distribution; (**b**) recording duration (in hours); (**c**) number of 30 s epochs per dataset.

Figure 2 shows the visualization that presents a comprehensive overview of three key aspects of EEG sleep datasets: sleep stage distribution, recording duration distribution, and number of epochs. The first section of the image shows the sleep stage distribution across the Sleep-EDF, MASS, and SHHS datasets, highlighting the proportion of Wake (W), N1, N2, N3, and REM stages. The second section features a box plot comparing the distribution of recording durations (in hours) for each dataset, illustrating the range and median values. The third section displays a bar chart that indicates the total number of 30 s epochs available in each dataset, with SHHS having the highest number, followed by MASS and Sleep-EDF. This visualization effectively captures the diversity and scale of the datasets, providing a clear understanding of their characteristics for EEG-based sleep stage classification research.

### 3.2. Data Preprocessing

Algorithm 1 shows the preprocessing of EEG signals which is a crucial step in preparing the data for accurate and reliable sleep stage classification using deep learning models. This process involves transforming raw EEG signals into a format that enhances the discriminative features necessary for effective classification.
**Algorithm 1** EEG Signal Preprocessing1: Input: Raw EEG signal «(t) $2:\;\mathrm{Output}:\;\mathrm{Normalized}\;\mathrm{time}-\mathrm{frequency}\;\mathrm{representations}\;S_{i}^{’}(m,f)$ 3: Step 1: Segment EEG Signal 4: for each segment i do $5:\;\;\;\;\;t_{i}\leftarrow \;\mathrm{start}\;\mathrm{time}\;\mathrm{of}\;\mathrm{segment}\;i$ $6:\;\;\;\;\;x_{i}\left(t\right)\leftarrow x\left(t\right)\;for\;t\in [t_{i},t_{i}+T]\;\;\mathrm{Segment}\;\mathrm{EEG}\;\mathrm{signal}\;\mathrm{into}\;30-\mathrm{second}\;\mathrm{epochs}$ 7: end for 8: Step 2: Time-Frequency Transformation $9:\;\mathrm{for}\;\mathrm{each}\;\mathrm{segment}\;x_{i\;}(t)\;\mathrm{do}$ 10: Option 1: Compute spectrogram using STFT: $11:\;S_{i}(m,\;f)\;\leftarrow \;\sum_{n=-\infty }^{\infty }x_{i}\left(n\right).w\left(n-m\right).\;e^{-j2\pi fn}=\;\;\mathrm{Calculate}\;\mathrm{STFT}\;\mathrm{for}\;\mathrm{each}\;\mathrm{segment}$ 12: Option 2: Compute wavelet coefficients: $13:\;\;\;\;\;\;W_{i}(a,\;b)\;\leftarrow CWT\;(x_{i}(t))\;\;\;\;\;\mathrm{Calculate}\;\mathrm{CWT}\;\mathrm{for}\;\mathrm{each}\;\mathrm{segment}$ 14: end for 15: Step 3: Normalization $16:\;\mathrm{for}\;\mathrm{each}\;\mathrm{time}-\mathrm{frequency}\;\mathrm{representation}\;S_{i}(m,\;f)\;\mathrm{or}\;W_{i}(a,b)\;\mathrm{do}$ 17: Compute mean μ and standard deviation σ $\;\;\;\;\;\;\;S_{i}^{’}\left(m,f\right)\leftarrow \;\frac{S_{i}\left(m,f\right)-\mu }{\sigma }$ 18: end for $19:\;\mathrm{Return}:\;\mathrm{Normalized}\;\mathrm{time}-\mathrm{frequency}\;\mathrm{representations}\;S_{i}^{’}\left(m,f\right)\;or\;W_{i}^{’}\left(a,b\right)$

**Figure 3:**
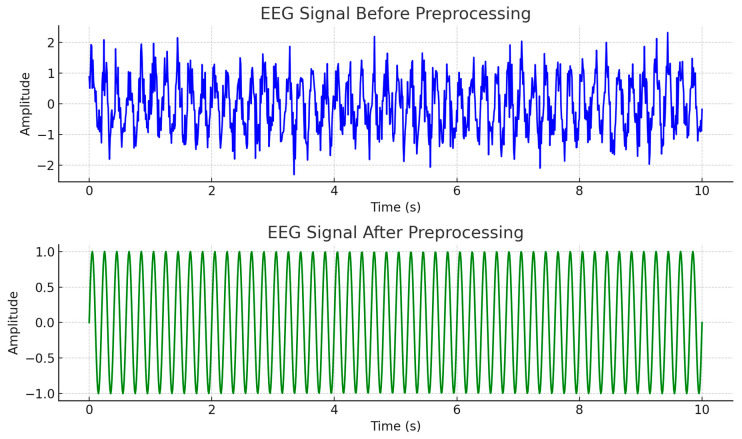
The EEG preprocessing process with output.

The preprocessing pipeline consists of three primary stages: signal cropping, spectrogram conversion, and normalization. Each of these steps is designed to systematically reduce noise, extract meaningful time–frequency information, and standardize the data for subsequent analysis. Figure 3 shows the outcomes of this phase.

The EEG signals are first segmented into smaller, more manageable time windows, typically 30 s epochs. Let the original EEG signal be denoted as *x*(*t*)*x*(*t*)*x*(*t*), where *ttt* represents time. The segmented signal for a window of TTT seconds is given by the following equation:

$$x_{i}\left(t\right)=x\left(t\right)for\;t\in [t_{i},t_{i}+T]$$

where $x_{i}\left(t\right)$ is the ith segment of the signal, and $t_{i}$ is the start time of the *i*th segment. The segmented EEG signals are then converted into the time–frequency domain using the Short-Time Fourier Transform (*STFT*).

$$STFT\{x_{i}(t)\}(m,f)=\sum_{n=-\infty }^{\infty }x_{i}\left(n\right)\cdot w\left(n-m\right).e^{-j2\pi fn}$$

where *m* is the time index, *f* is the frequency, w(n) is the window function, and *j* is the imaginary unit. The result of this step is a spectrogram $S_{i}(m,f)$ or wavelet coefficients matrix $W_{i}(a,b)$, representing the time–frequency characteristics of the *i*th segment. To standardize the data and reduce variability, normalization techniques such as z-score normalization or min–max scaling are applied to the spectrogram or wavelet coefficients.

$$S_{i}^{’}(m,f)=\frac{S_{i}\left(m,f\right)-\mu }{\sigma }$$

where μ is the mean, and *σ* is the standard deviation of the spectrogram values. These preprocessing steps ensure that the EEG data are transformed into a suitable format for further analysis, making the features more consistent and interpretable for downstream deep learning models.

### 3.3. Data Balancing

Data balancing is essential in every model, especially when dealing with imbalanced datasets where certain classes are significantly underrepresented. In such cases, a model trained on imbalanced data tends to be biased towards the majority class, leading to poor performance on minority classes. This step improves the model’s generalizability, fairness, and reliability, particularly in applications where minority classes are of high importance, such as sleep stage classification. In this work, class weights adjustment [xx] is used to address class imbalance by modifying the loss function during the training process of a machine learning model. Instead of altering the dataset itself, this method adjusts the penalty assigned to misclassifications of different classes, thereby encouraging the model to pay more attention to underrepresented classes.

In the proposed sleep stage classification model, if a dataset consists mostly of samples from the N2 sleep stage and very few from stages like REM or N3, the model may overwhelmingly predict N2, thereby ignoring the minority stages. For assigning the class weights, let N be the total number of samples in your dataset and $N_{c}$ be the number of samples in class c. The class weight $w_{c}$ for class *c* can be computed as follows:

$$w_{c}=\frac{N}{\left|C\right|\;{\cdot N}_{c}}$$

where |*C*| is the total number of classes. This formula ensures that minority classes receive a higher weight, making their misclassifications costlier during training. In the training of proposed sleep stage classification model, the standard loss function based on categorical cross-entropy is modified to include these class weights. The weighted loss function for a given sample i with true class label $y_{i}$ and predicted probability $p_{i}$ is then expressed as follows:

$$Weighted\;Loss_{i}=-w_{yi}\cdot log(p_{i},y_{i})$$

This adjustment ensures that the model does not disproportionately favor the majority class (e.g., N2) and instead strives to balance its predictions across all sleep stages. This approach is especially critical in applications where the minority stages, such as REM and N3, are of significant importance for understanding sleep patterns and diagnosing sleep disorders. By ensuring that your model considers all sleep stages equitably, data balancing allows for better-informed decisions and enhances the model’s overall performance across the board.

**Figure 4:**
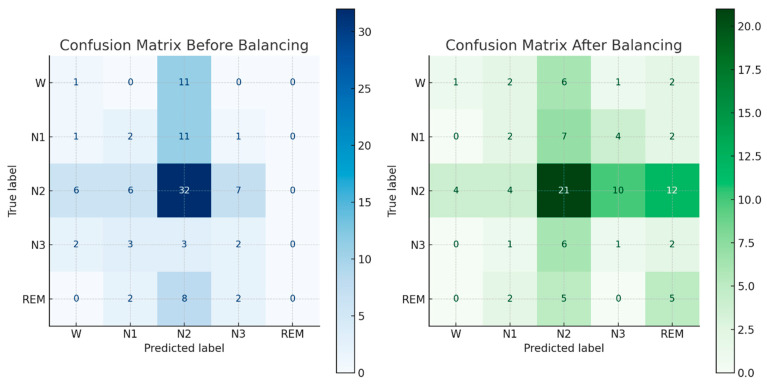
The confusion matrix showing biased and balanced position.

Figure 4 illustrates the impact of class weights adjustment on a sleep stage classification model through confusion matrices. Before balancing, the model shows a strong bias towards the majority class (N2), leading to a high number of correct predictions for this class but poor performance on the minority classes (W, N1, N3, and REM), as seen in the first confusion matrix. After applying class weights, the second confusion matrix reveals a more balanced distribution of correct predictions across all sleep stages. This improvement demonstrates that class weights adjustment effectively mitigates class imbalance, enhancing the model’s ability to accurately classify underrepresented sleep stages and leading to better overall performance.

### 3.4. Feature Extraction

Temporal Convolutional Networks (TCNs) are employed for feature extraction. TCNs are particularly suitable for handling sequential data like EEG signals because they can capture both short-term and long-term dependencies in the data. Unlike traditional convolutional neural networks (CNNs) that are typically applied to spatial data, TCNs are specifically designed to process time-series data, making them well-suited for sleep stage classification. A key characteristic of TCNs is the use of causal convolutions, which ensure that the predictions at any given time step depend only on the past and not on future data points, thereby preserving the temporal integrity of the EEG signals. The feature extraction process using TCNs involves several layers of convolutional operations, each designed to extract features at different levels of abstraction.

The process begins with the input EEG signal, denoted as x(t), which represents the signal amplitude at each time step t. The input signal is typically segmented into smaller time windows (e.g., 30 s epochs) for processing. The input signal x(t) is passed through a series of causal convolutional layers. A causal convolutional layer applies a convolution operation that only considers the current and previous time steps, ensuring that future data points do not influence the current output. The operation of a causal convolutional layer can be described as follows:

$$Conv_{i}\left(x\left(t\right)\right)=\sum_{k=0}^{K-1}w_{k}\cdot x(t-k)$$

where *K* represents the kernel size, $w_{k}$ represents the weights of the kernel at layer iii, and t−k ensures causality by using only past and current data points. The convolution operation extracts local features from the input signal by applying the learned weights $w_{k}$ to the input data. To capture longer-term dependencies without significantly increasing the computational cost, TCNs utilize dilated convolutions. Dilated convolutions introduce a dilation factor d, which determines the spacing between the kernel elements. This allows the network to have a larger receptive field, enabling it to capture temporal dependencies over longer time intervals. The operation of a dilated convolutional layer is given by the following equation:

$$Detailed\;Conv_{i}\left(x\left(t\right)\right)=\sum_{k=0}^{K-1}w_{k}\cdot x(t-dk)$$

In this equation, d is the dilation rate, and dk controls the spacing between the sampled input points. As the dilation factor increases, the receptive field expands, allowing the TCN to capture more extensive patterns in the EEG signal. To facilitate the training of deep networks and prevent the vanishing gradient problem, TCNs often incorporate residual connections between layers. A residual connection allows the input to bypass one or more convolutional layers and be added directly to the output. The operation of a residual connection can be expressed as follows:

$$Output_{i}=Conv_{i}(x(t))+x(t)$$

This addition helps to preserve the original input information while allowing the network to learn the residual mappings, leading to more stable and efficient training.

After passing through multiple layers of causal and dilated convolutions, the network produces a set of feature maps that represent the learned patterns in the EEG signal. These feature maps are then aggregated to form a final feature vector f, which serves as the input to the classification layers of the model. The aggregated feature vector can be represented as follows:

$$f=[Conv_{n}(x(t)),Dilated\;Conv_{n}(x(t)),\ldots \;]$$

where *n* is the number of convolutional layers in the TCN. The feature vector f encapsulates information from both local and global patterns in the EEG signal, providing a comprehensive representation for classification.

**Figure 5:**
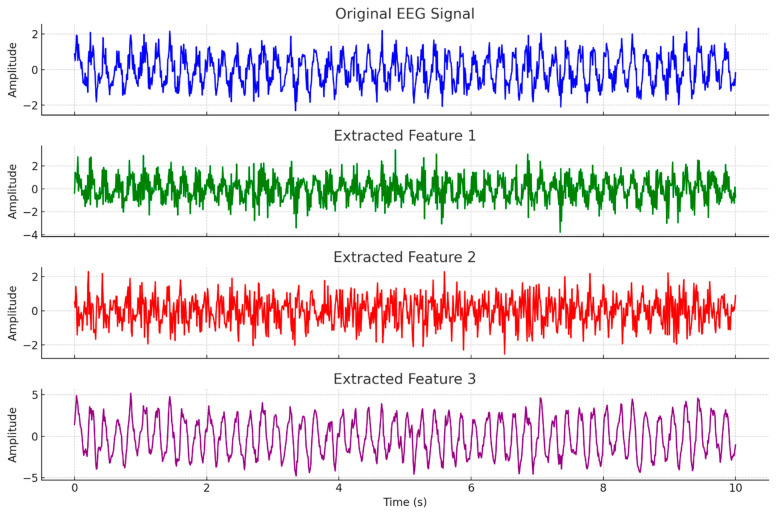
The extracted feature snippet.

Figure 5 illustrates the process of feature extraction from an original EEG signal using a Temporal Convolutional Network (TCN)-like approach. The first plot shows the original EEG signal, characterized by a 5 Hz sine wave with added noise, which represents the raw input data. The subsequent plots display the extracted features, each derived from the original signal through convolutional operations that mimic the layers in a TCN. These extracted features capture different patterns and characteristics of the EEG signal, highlighting various aspects such as local variations and temporal dependencies.

### 3.5. Final Sleep Stage Classification

After extracting features using the Temporal Convolutional Network (TCN), the next step is to perform the final classification using a Multilayer Perceptron (MLP) trained with Stochastic Gradient Descent (SGD). Algorithm 2 presents the workflow of final classification where the feature vector f produced by the TCN, which encapsulates the temporal patterns from the EEG signals, is used as the input to the MLP. The MLP consists of multiple fully connected layers, where each hidden layer performs a linear transformation of the input followed by a non-linear ReLU [xx] activation function. This process is represented by the following equation:

$$h_{i}=\sigma (W_{i}h_{i-1}+b_{i})$$

where $W_{i}$ and $b_{i}$ are the weights and biases of the layer, and *σ* denotes the ReLU activation. The final hidden layer is connected to the output layer, which computes logits for each sleep stage using the following equation:

$$z=W_{out}h_{last}+b_{out}\;$$

**Algorithm 2** Final Classification Using Multilayer Perceptron (MLP) Trained with Stochastic Gradient Descent (SGD) 1: Input: Feature Vector f from TCN, True Labels y $2:\;\mathrm{Output}:\;\mathrm{Predicted}\;\mathrm{Sleep}\;\mathrm{Stage}\;\mathrm{Labels}\;\hat{y}\;$ 3: Step 1: Initialize MLP 4: Define MLP structure with multiple fully connected layers $5:\;\mathrm{Initialize}\;\mathrm{weights}\;W_{i}\;\mathrm{and}\;\mathrm{biases}\;b_{i}\;\mathrm{for}\;\mathrm{each}\;\mathrm{layer}$ 6: Step 2: Forward Pass 7: for each training iteration t do 8:     for each layer i: $9:\;\;\;\;\;\;h_{i}\;\leftarrow \;\sigma \left(W_{i}h_{i-1}+b_{i}\right)$ 10: end for 11: Compute output logits: $12:\;\leftarrow \;W_{out}h_{last}+b_{out}$ 13: Step 3: Compute Cross-Entropy Loss $14:\;L\left(y,z\right)\leftarrow -\sum_{i=1}^{C}y_{i}\log\left(\frac{\exp\left(z_{i}\right)}{\sum_{j-1}^{c}\exp\left(z_{j}\right)}\right)$ 15: Step 4: Backpropagation and Optimization 16: for each training iteration t do $17:\;\;\;\;\;\;\mathrm{Compute}\;\mathrm{gradients}\;\mathrm{of}\;L\;\mathrm{with}\;\mathrm{respect}\;\mathrm{to}\;W_{i}\;\mathrm{and}\;b_{i}$ $18:\;\;\;\;\;\;\mathrm{Update}\;W_{i}\;\leftarrow \;W_{i}-n\frac{\sigma L}{\sigma W_{i}}$ $19:\;\;\;\;\;\;\mathrm{Update}\;b_{i}\;\leftarrow \;b_{i}-n\frac{\sigma L}{\sigma b_{i}}$ 20: end for 21: Step 5: Final Classification 22: for each new EEG feature vector f do 23: Perform forward pass through MLP to compute logits z $24:\;\;\;\;\;ŷ\leftarrow argmaxz_{i}$ 25: end for 26: Return: Predicted Sleep Stage Labels ŷ

Training the MLP involves minimizing a loss function, specifically cross-entropy, that quantifies the difference between the predicted logits and the true sleep stage labels. The cross-entropy loss is expressed as follows:

$$L\left(y,z\right)=-\sum_{i=1}^{C}y_{i}\log\left(\frac{\exp\left(z_{i}\right)}{\sum_{j=1}^{C}\exp\left(z_{i}\right)}\right)$$

where *y* is the one-hot encoded true label, $z_{i}$ represents the logits for class *i*, and *C* is the number of classes (sleep stages). During training, the MLP learns by iteratively updating its weights and biases using SGD. In each iteration, the model performs forward propagation to compute the output logits, calculates the cross-entropy loss, and then back-propagates the error to compute gradients of the loss with respect to the weights and biases. These gradients are used to update the weights and biases as follows:

$$W_{i}\leftarrow W_{i}-\eta \frac{\partial L}{\partial W_{i}}$$

$$b_{i}\leftarrow b_{i}-\eta \frac{\partial L}{\partial b_{i}}$$

where *η* is the learning rate. Once training is complete, the MLP can be used for inference by passing new EEG feature vectors through the network to generate logits. The predicted sleep stage is determined by selecting the class with the highest logit value:

$$Predicted\;class=arg\underset{i}{\max}z_{i}$$

## 4. Experimental Results and Evaluation

The experimental evaluation of the proposed SLA-MLP is presented in this section. The proposed sleep stage classification model requires significant computational resources due to the complexity of the Temporal Convolutional Network (TCN) and Multilayer Perceptron (MLP) architectures and the large size of the datasets. Training the model involves extensive computations for convolutions, feature extraction, and iterative weight updates. The experiments were conducted using an NVIDIA RTX 3080 GPU, an Intel Core i7 multi-core CPU, and 32 GB of RAM, which facilitated efficient handling of computationally intensive tasks.

The hyperparameter selection process was conducted using grid search and manual tuning to optimize model performance. The optimal learning rate was 0.001, with a batch size of 32 for efficient training. A kernel size of 3 and a dilation rate of 2 were chosen for convolutional layers to effectively capture temporal dependencies. Dropout was set at 0.3, with L2 regularization using a weight decay of 0.0001 to prevent overfitting. MLP layers were configured with 128 neurons per hidden layer, balancing complexity and performance. These values were finalized based on validation performance and computational efficiency. The details of performance metrics, base line methods, and comparative analysis are presented in the subsections below.

### 4.1. Performance Metrics

Table 2 shows the list of performance metrics that have been used as standard evaluation measures for SESDP.

### 4.2. Baseline Models

Three different baseline approaches have been used for the evaluation of the proposed model:

Kong et al. [23]: proposed a novel neural architecture search (NAS) framework for EEG-based sleep stage classification, leveraging bilevel optimization approximation. The framework employs search space approximation and regularization with shared parameters among cells to optimize the model;

Phan et al. [24]: the study introduces L-SeqSleepNet, a new deep learning model designed to efficiently handle long sequence modeling for sleep staging. Unlike existing models, L-SeqSleepNet incorporates whole-cycle sleep information, achieving state-of-the-art performance across various EEG setups, including scalp EEG, in-ear EEG, and around-the-ear EEG.

Heng et al. [25]: an automatic end-to-end sleep stage classification method is proposed, focusing on single-channel sleep EEG signals. The method uses a convolutional neural network (CNN) with a squeeze-and-excitation block (SE-Block) to enhance feature learning and address the long-term time series problem in sleep EEG signals.

## 5. Results

**Figure 6:**
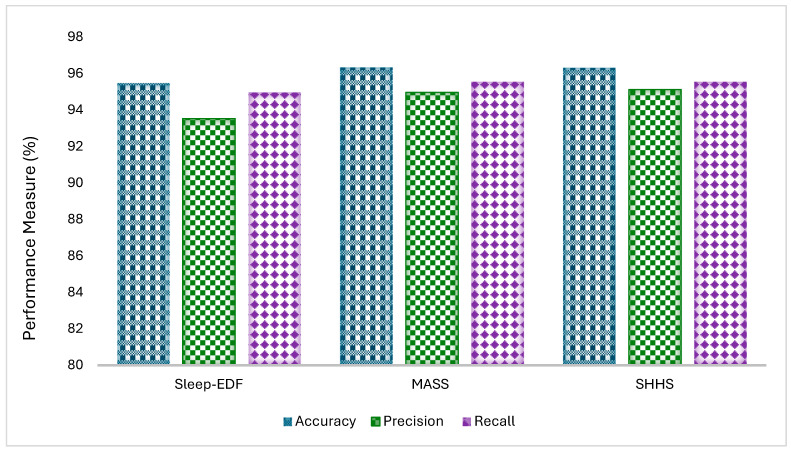
Experimental results on Sleep-EDF, MASS, and SHHS datasets.

The proposed Sleep Stage Analysis with Multilayer Perceptron (SLA-MLP) model demonstrated strong performance across three widely used sleep datasets: Sleep-EDF, MASS, and SHHS as shown in Figure 6. The model achieved high accuracy, with results of 95.48% for the Sleep-EDF dataset, 96.34% for the MASS dataset, and 96.32% for the SHHS dataset. These accuracy scores indicate the model’s robust ability to classify sleep stages accurately across different datasets. Additionally, the model performed well in terms of precision, which measures the proportion of true positive predictions out of all positive predictions, achieving 93.52%, 94.98%, and 95.13% precision for the Sleep-EDF, MASS, and SHHS datasets, respectively. The recall, which evaluates the model’s ability to correctly identify all actual positive instances, was also high, with the model reaching 94.97% recall for the Sleep-EDF dataset, 95.56% for the MASS dataset, and 95.56% for the SHHS dataset. These results highlight the effectiveness and consistency of the SLA-MLP model in accurately classifying sleep stages, making it a valuable tool for sleep research and clinical applications.

**Figure 7:**
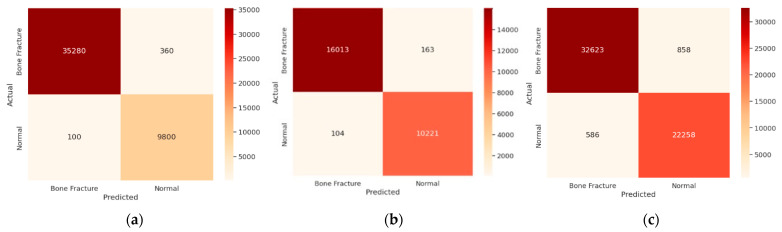
Confusion matrix of Deep-I, Deep-II, and Deep-III. (**a**) Confusion matrix of Deep-I; (**b**) confusion matrix of Deep-II; (**c**) confusion matrix of Deep-III.

In another experiment, confusion matrices were employed to evaluate the effectiveness of the proposed approach in distinguishing between normal and bone fracture instances, as illustrated in Figure 7. The model demonstrated a notable average accuracy of 96.12% across all datasets, indicating a high true positive rate while maintaining a low false positive rate across various classification thresholds.

**Figure 8:**
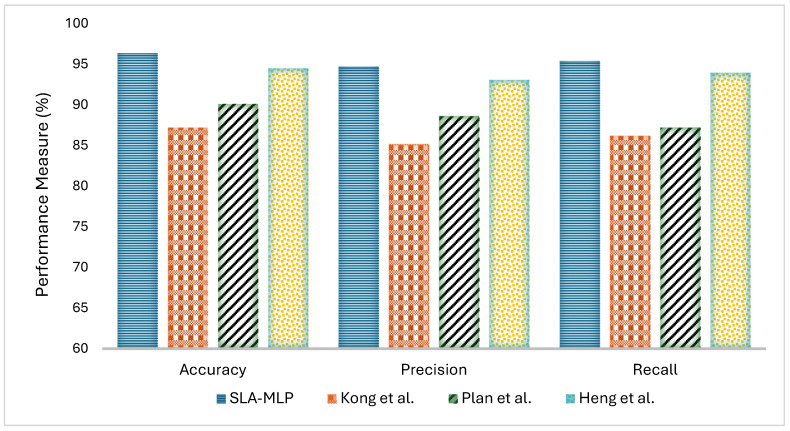
Comparison with baseline approaches in terms of accuracy, precision, and recall [23,24,25].

The Sleep Stage Analysis with Multilayer Perceptron (SLA-MLP) model demonstrates superior performance when compared against baseline models in the literature—specifically, Kong et al., Plan et al., and Heng et al.—as shown in Figure 8. The SLA-MLP model achieved an impressive accuracy of 96.37%, surpassing Kong et al. (87.23%), Plan et al. (90.15%), and Heng et al. (94.49%). This high accuracy underscores the advanced capabilities of the SLA-MLP model in accurately classifying sleep stages, benefiting from sophisticated preprocessing and feature extraction methods. In terms of precision, the SLA-MLP model also leads with a score of 94.7%, which is a significant improvement over Kong et al. at 85.18% and Plan et al. at 88.62%. It also slightly surpasses Heng et al., who achieved a precision of 93.05%. This metric reflects the SLA-MLP model’s ability to minimize false positives, a crucial factor in medical diagnostics where precision is as important as accuracy. Recall, a critical measure of a model’s ability to identify all relevant cases within a dataset, saw the SLA-MLP model performing at 95.4%, again outperforming Kong et al. (86.21%), Plan et al. (87.2%), and Heng et al. (93.94%). The high recall rate of the SLA-MLP model ensures that it effectively captures the majority of true sleep stage cases, minimizing the risk of false negatives, which can be particularly detrimental in clinical settings.

The log loss comparison of the proposed Sleep Stage Analysis with Multilayer Perceptron (SLA-MLP) model against baseline models, including those by Kong et al., Plan et al., and Heng et al., further establishes its superiority in classification accuracy across multiple datasets (Table 3). On the Sleep-EDF dataset, the SLA-MLP model achieved the lowest log loss of 0.350, compared to Kong et al. at 0.450, Plan et al. at 0.520, and Heng et al. at 0.390. This trend of superior performance continues with the MASS dataset, where the SLA-MLP model registered a log loss of 0.345, outperforming Kong et al. (0.460), Plan et al. (0.530), and Heng et al. (0.400). Similarly, on the SHHS dataset, the proposed model’s log loss was 0.321, significantly better than Kong et al. (0.465), Plan et al. (0.525), and Heng et al. (0.395). These results highlight the SLA-MLP model’s consistent ability to minimize the uncertainty in predicting sleep stages, confirming its robustness and reliability as a diagnostic tool for sleep stage classification. This exceptional performance across diverse datasets underscores the model’s potential to facilitate more accurate and reliable sleep stage assessments in clinical and research settings.

Despite the proposed model’s advancements in sleep stage classification, this work has several limitations. First, the reliance on specific datasets such as Sleep-EDF, MASS, and SHHS restricts the generalizability of the model to other populations or datasets with different demographic or clinical characteristics. Additionally, the computational complexity of the Temporal Convolutional Networks (TCNs) and Multilayer Perceptron (MLP) models may also pose challenges for real-time or resource-constrained applications, such as wearable devices. Furthermore, the use of class weights for data balancing, while effective, might not fully address extreme class imbalances in other datasets, potentially impacting performance on minority sleep stages

## 6. Conclusions and Future Work

The analysis and classification of sleep stages are crucial for diagnosing sleep disorders and understanding overall sleep health. This process is particularly important for psychologists and psychiatrists working in mental health research, as it enables faster screening of sleep disorders. Traditional methods, including manual scoring and basic machine learning approaches, often fall short due to subjective biases, scalability issues, and limited accuracy. While deep learning models have significantly enhanced sleep stage classification, challenges like overfitting, computational inefficiencies, and imbalanced datasets remain prevalent. This study introduced the Sleep Stage Analysis with Multilayer Perceptron (SLA-MLP) model, which leverages advanced deep learning techniques to improve sleep stage classification using EEG signals. Key contributions of our approach include robust preprocessing steps such as spectrogram conversion and signal normalization, effective data balancing strategies, feature extraction using Temporal Convolutional Networks (TCNs), and precise classification through a Multilayer Perceptron (MLP). The SLA-MLP model demonstrated superior performance compared to traditional and existing deep learning methods by addressing key limitations such as overfitting and dataset imbalances. Future research will focus on integrating multimodal physiological signals, such as EMG and EOG, to improve classification accuracy further. Additionally, future work should focus on optimizing lightweight models for real-time applications, enhancing data balancing techniques, and incorporating explainable AI for improved interpretability.

## Figures and Tables

**Table 1:** Literature review table summarizing existing models.

Refs.	Methodology	Dataset	Accuracy	Key Limitations
[17]	ResNet architecture for classifying sleep states using EEG and EMG signals	Mice data + Recovery sleep.	97% overall accuracy; 96% F1 score.	Limited to mouse data, which may not generalize to human sleep studies. Small and diverse training dataset may affect robustness.
[18]	Convolutional neural network (CNN)	EEG signals from six channels.	99.39% accuracy for channel C4-A1; other channels above 98.5%.	High accuracy is specific to certain channels. Requires significant computational resources for 2D CNN.
[19]	Convolutional neural network with long short-term memory (LSTM)	Sleep EEG recordings from various subjects.	95% accuracy with improved temporal consistency.	Complex model architecture leads to high computational cost. May require extensive tuning for different datasets.
[20]	Autoencoder	Large-scale EEG dataset from multiple sleep studies.	93% accuracy with reduced feature dimensionality.	Performance may degrade with noisy or incomplete data. Limited interpretability of extracted features.
[21]	SleepNet with convolutional layers	EEG data from clinical sleep studies.	96.5% accuracy with improved stage discrimination.	Higher computational requirements due to attention mechanisms. May not perform well with highly variable sleep data.
[22]	Wavelet transform combined with deep learning	Multi-channel EEG data	94% accuracy with enhanced feature representation.	Complexity of wavelet transform adds to processing time. May struggle with real-time analysis in practical applications.

**Table 2:** List of performance metrics.

S. No.	Metrics	Formula
**1**	The primary metrics are as follows: **accuracy**, which indicates the proportion of correctly classified samples out of the total sample size, calculated using true positives (TP), true negatives (TN), false positives (FP), and false negatives (FN). **precision**, which measures the proportion of true positive predictions among all positive predictions made via the model. **recall**, or sensitivity, which reflects the proportion of true positive samples correctly identified via the model out of all actual positive samples. The **F1-Score**, which provides a balance between precision and recall, serving as the harmonic mean of these two metrics.	$\begin{array}{c} Accuracy=\frac{TP+TN}{TP+TN+FP+FN}\\ precision=\frac{TP}{TP+FP}\\ recall=\frac{TP}{TP+FN}\;F1\;Score=2\times \frac{P\times R}{P+R} \end{array}$
**2**	MCC is a robust metric for evaluating the quality of binary classifications, as it takes into account all four components of the confusion matrix. MCC ranges from −1 to 1, where 1 represents perfect classification, 0 corresponds to random prediction, and −1 indicates total misclassification.	$MCC=\frac{TP\times TN-FP\times FN}{\sqrt{\left(TP+FP)(TP+FN)(TN+FP)(TN+FN\right)}}$
**3**	Log loss assesses the performance of a classification model by penalizing incorrect predictions according to the predicted probabilities.	$Log\;Los=-\frac{1}{N}\sum_{i=1}^{n}\left(y_{i}log(p_{i})+(1-y_{i})log(1-p_{i})\right)$

**Table 3:** Log loss comparison of proposed model with baselines.

Dataset	Kong et al. [23]	Plan et al. [24]	Heng et al. [25]	SLA-MLP (Proposed Model)
Sleep-EDF	0.450	0.520	0.390	0.350
MASS	0.460	0.530	0.400	0.345
SHHS	0.465	0.525	0.395	0.321

## Data Availability

The original contributions presented in the study are included in the article, further inquiries can be directed to the corresponding authors.
